# Supplementary material for: Electromyographic biofeedback therapy for improving limb function after stroke: A systematic review and meta-analysis
Source: PLoS One. 2024 Jan 11;19(1):e0289572. doi: 10.1371/journal.pone.0289572 (PMC10783731; doi:10.1371/journal.pone.0289572)
Supplement: S1 Fig — (DOC) [file pone.0289572.s002.doc]

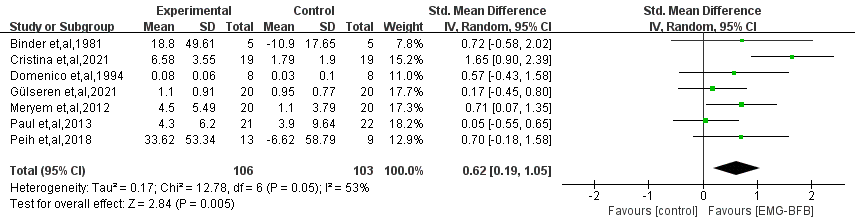


**Figures S1:** Forest plot of sensitivity analysis by excluding studies with a high risk of bias.The pooled effect size measures limb function improvement post electromyographic biofeedback therapy compared to control.
