# Supplementary material for: Electromyographic biofeedback therapy for improving limb function after stroke: A systematic review and meta-analysis
Source: PLoS One. 2024 Jan 11;19(1):e0289572. doi: 10.1371/journal.pone.0289572 (PMC10783731; doi:10.1371/journal.pone.0289572)
Supplement: S3 Fig — Abbreviation: RoB2, Risk of Bias version 2. (DOC) [file pone.0289572.s004.doc]

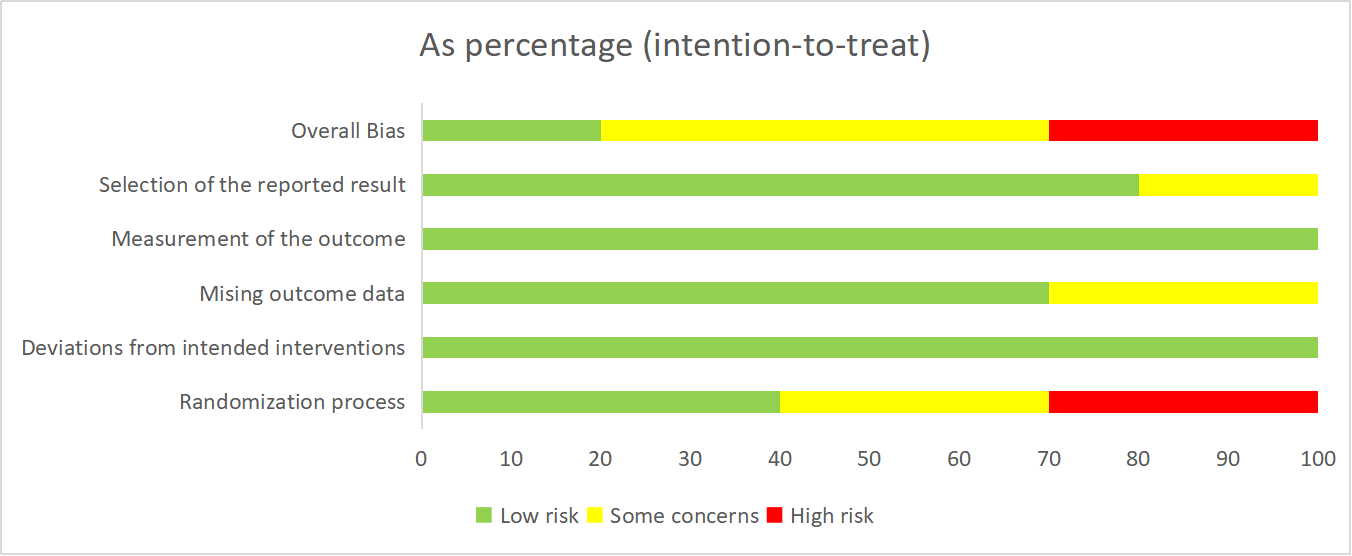


**Figures S3:** Percentage graph of risk of bias across domains. Abbreviation: RoB2, Risk of Bias version 2.
