# Supplementary material for: Electromyographic biofeedback therapy for improving limb function after stroke: A systematic review and meta-analysis
Source: PLoS One. 2024 Jan 11;19(1):e0289572. doi: 10.1371/journal.pone.0289572 (PMC10783731; doi:10.1371/journal.pone.0289572)
Supplement: S4 Fig — (DOC) [file pone.0289572.s005.doc]

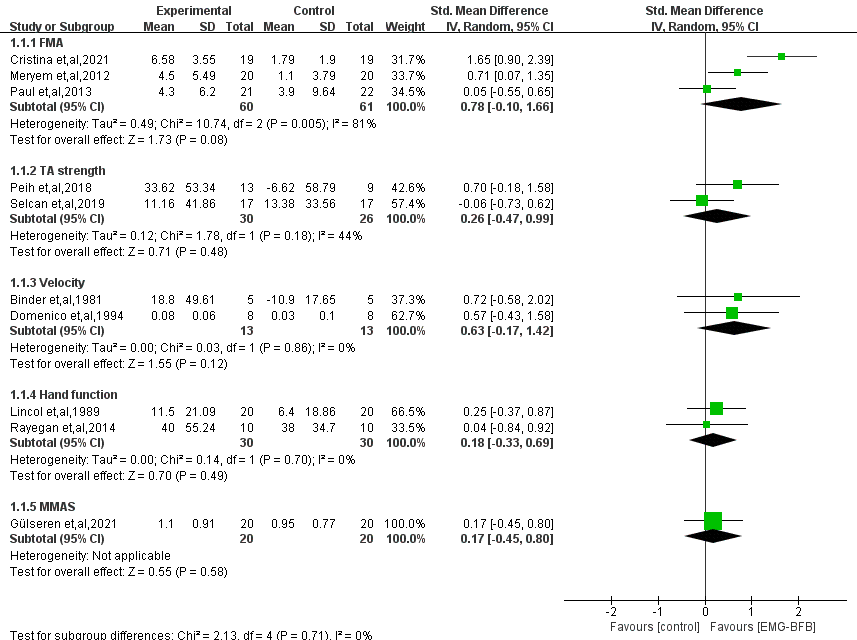


**Figures S4:** Pooled effects of different limb function scales after electromyographic biofeedback therapy as compared with control interventions. SMD, standardized mean difference. Weights and between-subgroup heterogeneity test are from random-effects model.
