# Supplementary material for: Electromyographic biofeedback therapy for improving limb function after stroke: A systematic review and meta-analysis
Source: PLoS One. 2024 Jan 11;19(1):e0289572. doi: 10.1371/journal.pone.0289572 (PMC10783731; doi:10.1371/journal.pone.0289572)
Supplement: S5 Fig — (DOC) [file pone.0289572.s006.doc]

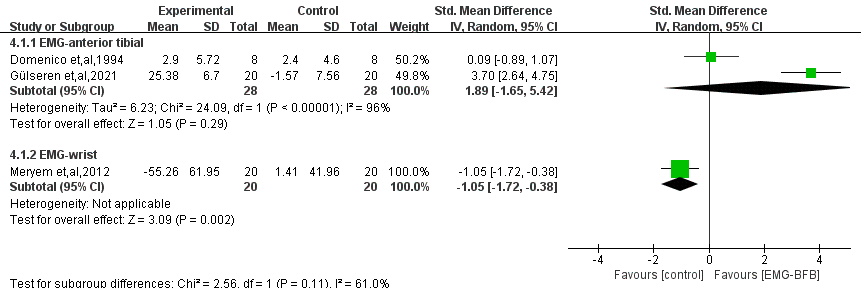


**Figures S5:**  Secondary outcomes: forest plot of relevant muscle EMG values. SMD, standardized mean difference. Tests for weight and heterogeneity between subgroups were derived from random effects models.
