# Supplementary material for: Electromyographic biofeedback therapy for improving limb function after stroke: A systematic review and meta-analysis
Source: PLoS One. 2024 Jan 11;19(1):e0289572. doi: 10.1371/journal.pone.0289572 (PMC10783731; doi:10.1371/journal.pone.0289572)
Supplement: S7 Fig — (DOC) [file pone.0289572.s008.doc]

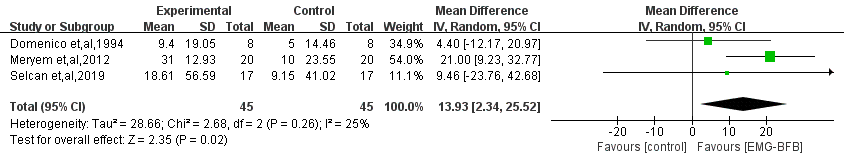


**Figure S7:** Forest plot of subgroup sensitivity analysis by excluding studies with a high risk of bias. The pooled effect size measures Barthel Index improvement post electromyographic biofeedback therapy compared to control.
