# Supplementary figures and images for: Electromyographic biofeedback therapy for improving limb function after stroke: A systematic review and meta-analysis
Source: PLoS One. 2024 Jan 11;19(1):e0289572. doi: 10.1371/journal.pone.0289572 (PMC10783731; doi:10.1371/journal.pone.0289572)

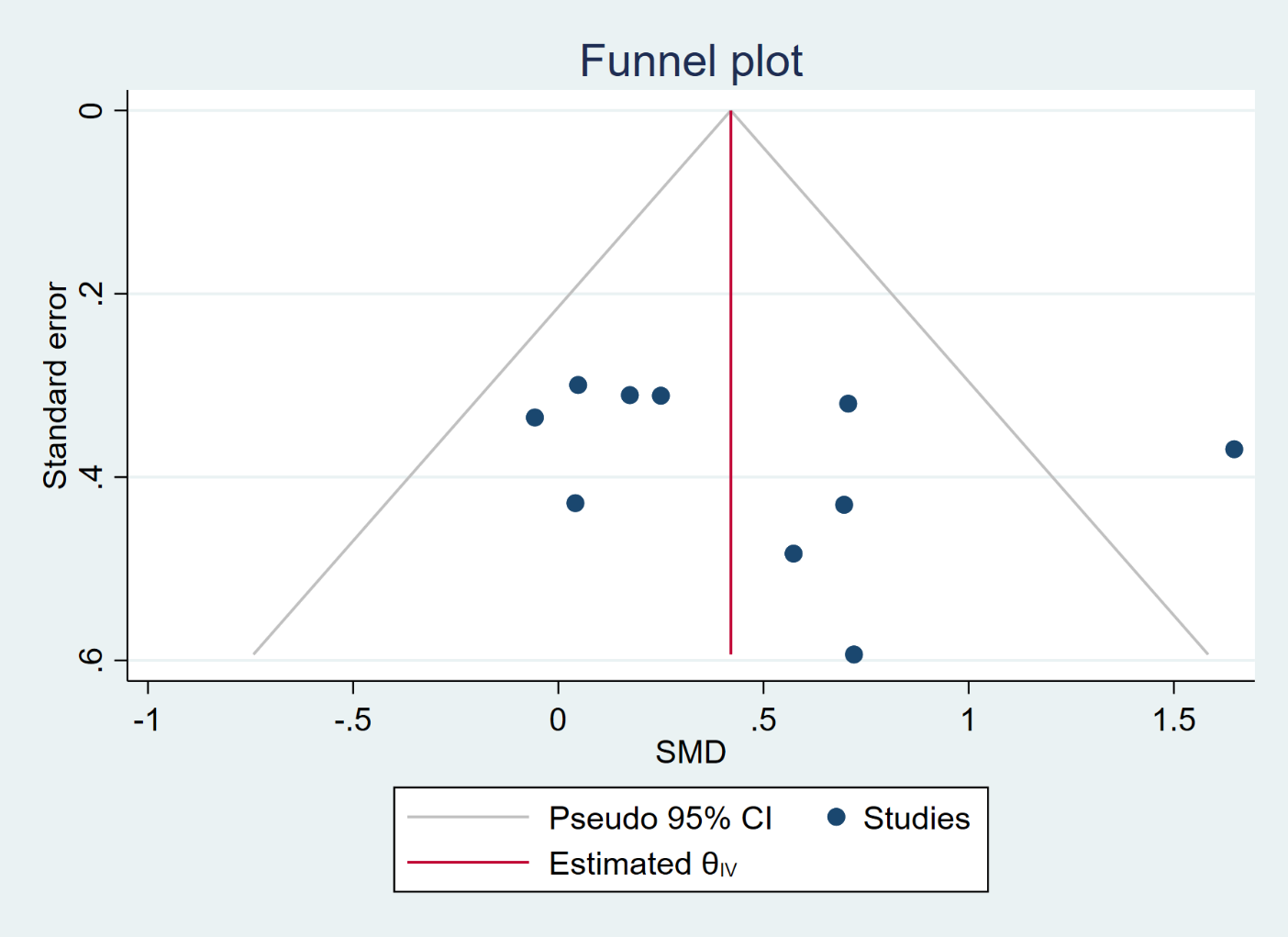


**Figures S8:** Funnel plot of scale scores based on the limb function evaluation index.

Supplement: S8 Fig — (DOC) [file pone.0289572.s009.doc]
