# Supplementary material for: Electromyographic biofeedback therapy for improving limb function after stroke: A systematic review and meta-analysis
Source: PLoS One. 2024 Jan 11;19(1):e0289572. doi: 10.1371/journal.pone.0289572 (PMC10783731; doi:10.1371/journal.pone.0289572)
Supplement: S1 Table — (DOC) [file pone.0289572.s010.doc]

Table 1. Literature search strategy in different electronic databases

| Database | Time | Strategy | Number of Records |
| --- | --- | --- | --- |
| Pubmed | 2022.05.01 | (("randomized controlled trial"[Publication Type] OR "controlled clinical trial"[Publication Type] OR "randomized"[Title/Abstract] OR "placebo"[Title/Abstract] OR "clinical trials as topic"[MeSH Terms:noexp] OR "randomly"[Title/Abstract] OR "trial"[Title]) NOT ("animals"[MeSH Terms] NOT ("humans"[MeSH Terms] AND "animals"[MeSH Terms]))) AND (("Cerebrovascular Disorders"[MeSH Terms] OR "Basal Ganglia Cerebrovascular Disease"[MeSH Terms] OR "Brain Ischemia"[MeSH Terms] OR "Carotid Artery Diseases"[MeSH Terms] OR "Intracranial Arterial Diseases"[MeSH Terms] OR "Intracranial Arteriovenous Malformations"[MeSH Terms] OR "Intracranial Embolism and Thrombosis"[MeSH Terms] OR "Intracranial Hemorrhages"[MeSH Terms] OR "Stroke"[MeSH Terms] OR "Brain Infarction"[MeSH Terms] OR ("Brain Injuries"[MeSH Terms] OR "brain injury, chronic"[MeSH Terms]) OR ("stroke*"[Title/Abstract] OR "cva"[Title/Abstract] OR "poststroke"[Title/Abstract] OR "post-stroke"[Title/Abstract]) OR ("cerebrovasc*"[Title/Abstract] OR "cerebral vascular"[Title/Abstract]) OR (("cerebral"[Title/Abstract] OR "cerebellar"[Title/Abstract] OR "brain*"[Title/Abstract] OR "vertebrobasilar"[Title/Abstract]) AND ("infarct*"[Title/Abstract] OR "ischemi*"[Title/Abstract] OR "ischaemi*"[Title/Abstract] OR "thrombo*"[Title/Abstract] OR "emboli*"[Title/Abstract] OR "apoplexy"[Title/Abstract])) OR (("cerebral"[Title/Abstract] OR "brain"[Title/Abstract] OR "subarachnoid"[Title/Abstract]) AND ("haemorrhage"[Title/Abstract] OR "hemorrhage"[Title/Abstract] OR "haematoma"[Title/Abstract] OR "hematoma"[Title/Abstract] OR "bleed*"[Title/Abstract])) OR ("Hemiplegia"[MeSH Terms] OR "Paresis"[MeSH Terms]) OR ("paretic"[Title/Abstract] OR "Paresis"[Title/Abstract] OR "hemipleg*"[Title/Abstract] OR "brain injur*"[Title/Abstract]) OR "gait disorders, neurologic"[MeSH Terms]) AND ("Electromyography"[MeSH Terms] OR ("biofeedback, psychology"[MeSH Terms] OR ("Feedback"[MeSH Terms] OR "feedback, psychological"[MeSH Terms])) OR ("electromyograph*"[Title/Abstract] OR "electromyogram*"[Title/Abstract] OR "emg"[Title/Abstract]) OR ("biofeedback"[Title/Abstract] OR "Feedback"[Title/Abstract]))) | 1164 |
| Embase | 2022.05.01 | #29 #27 AND #28  #28 'crossover procedure':de OR 'double-blind procedure':de OR 'randomized controlled trial':de OR 'single-blind procedure':de OR random*:de,ab,ti OR factorial*:de,ab,ti OR crossover*:de,ab,ti OR ((cross NEXT/1 over*):de,ab,ti) OR placebo*:de,ab,ti OR ((doubl* NEAR/1 blind*):de,ab,ti) OR ((singl* NEAR/1 blind*):de,ab,ti) OR assign*:de,ab,ti OR allocat*:de,ab,ti OR volunteer*:de,ab,ti  #27 #20 AND #26  #26 #21 OR #22 OR #23 OR #24 OR #25  #25 electromyograph*:ab,ti OR electromyogram*:ab,ti OR emg*:ab,ti OR biofeedback:ab,ti OR feedback:ab,ti  #24 'psychological feedback'/exp  #23 'feedback system'/exp  #22 'biofeedback'/exp  #21 'electromyography'/exp  #20 #1 OR #2 OR #3 OR #4 OR #5 OR #6 OR #7 OR #8 OR #9 OR #10 OR #11 OR #12 OR #13 OR #14 OR #17 OR #18 OR #19  #19 'neurologic gait disorder'/exp  #18 hempar*:ab,ti OR paretic:ab,ti OR paresis:ab,ti OR hemipleg*:ab,ti OR 'brain injur*':ab,ti  #17 #15 OR #16  #16 'paresis'/exp  #15 'hemiplegia'/exp  #14 (cerebral:ab,ti OR brain:ab,ti OR subarachnoid:ab,ti) AND (haemorrhage:ab,ti OR hemorrhage:ab,ti OR haematoma:ab,ti OR hematoma:ab,ti OR bleed*:ab,ti)  #13 (cerebral:ab,ti OR cerebellar:ab,ti OR brain*:ab,ti OR vertebrobasilar:ab,ti) AND (infarct*:ab,ti OR ischemi*:ab,ti OR ischaemi*:ab,ti OR thrombo*:ab,ti OR emboli*:ab,ti OR apoplexy:ab,ti)  #12 stroke*: ab,ti OR cva:ab,ti OR poststroke:ab,ti OR post‐stroke:ab,ti OR cerebrovasc*:ab,ti OR 'cerebral vascular':ab,ti  #11 'brain injury'/exp  #10 'brain infarction'/exp  #9 'cerebrovascular accident'/exp  #8 'brain hemorrhage'/exp  #7 'thromboembolism'/exp  #6 'brain arteriovenous malformation'/exp  #5 'cerebral artery disease'/exp  #4 'carotid artery disease'/exp  #3 'brain ischemia'/exp  #2 'basal ganglion hemorrhage'/exp  #1 'cerebrovascular disease'/exp | 2418 |
| Cochrane Liabray | 2022.05.01 | #1 MeSH descriptor: [Cerebrovascular Disorders] explode all trees  #2 MeSH descriptor: [Brain Ischemia] explode all trees  #3 MeSH descriptor: [Basal Ganglia Cerebrovascular Disease] explode all trees  #4 MeSH descriptor: [Carotid Artery Diseases] explode all trees  #5 MeSH descriptor: [Intracranial Arterial Diseases] explode all trees  #6 MeSH descriptor: [Intracranial Arteriovenous Malformations] explode all trees  #7 MeSH descriptor: [Intracranial Embolism and Thrombosis] explode all trees  #8 MeSH descriptor: [Intracranial Hemorrhages] explode all trees  #9 MeSH descriptor: [Stroke] explode all trees  #10 MeSH descriptor: [Brain Infarction] explode all trees  #11 #1 OR #2 OR #3 OR #4 OR #5 OR #6 OR #7 OR #8 OR #9 OR #10  #12 MeSH descriptor: [Brain Injuries] explode all trees  #13 MeSH descriptor: [Brain Injury, Chronic] explode all trees  #14 #12 OR #13  #15 (stroke* or cva or poststroke or post‐stroke):ti,ab,kw OR (cerebrovasc* or cerebral vascular):ti,ab,kw  #16 (cerebral or cerebellar or brain* or vertebrobasilar): ti,ab,kw AND (infarct* or ischemi* or ischaemi* or thrombo* or emboli* or apoplexy):ti,ab,kw  #17 (cerebral or brain or subarachnoid): ti,ab,kw AND (haemorrhage or hemorrhage or haematoma or hematoma or bleed*):ti,ab,kw  #18 MeSH descriptor: [Hemiplegia] explode all trees  #19 MeSH descriptor: [Paresis] explode all trees  #20 #18 OR #19  #21 (hempar* or paretic or paresis or hemipleg* or brain injur*):ti,ab,kw  #22 MeSH descriptor: [Gait Disorders, Neurologic] explode all trees  #23 #11 OR #14 OR #15 OR #16 OR #17 OR #20 OR #21 OR #22  #24 MeSH descriptor: [Electromyography] explode all trees  #25 MeSH descriptor: [Biofeedback, Psychology] explode all trees  #26 MeSH descriptor: [Feedback] explode all trees  #27 MeSH descriptor: [Feedback, Psychological] explode all trees  #28 #25 OR #26 OR #27  #29 (electromyograph* or electromyogram* or EMG*):ti,ab,kw OR (biofeedback or feedback):ti,ab,kw  #30 #24 OR #28 OR #29  #31 #23 AND #30 | 2499 |
| PEDro | 2022.05.01 | #1 "stroke*"[Title/Abstract]  #2 "poststroke"[Title/Abstract]  #3 "cerebral vascular"[Title/Abstract]  #4"cerebral"[Title/Abstract]  #5 #1 OR #2 OR #3 OR #4  #6 "electromyographic"[Title/Abstract]  #7 "emg"[Title/Abstract]  #8"biofeedback"[Title/Abstract]  #9"Feedback"[Title/Abstract]  #10 #6 OR #7 OR #8 OR #9  #11 #5 AND #10 | 10 |
