# Supplementary material for: Electromyographic biofeedback therapy for improving limb function after stroke: A systematic review and meta-analysis
Source: PLoS One. 2024 Jan 11;19(1):e0289572. doi: 10.1371/journal.pone.0289572 (PMC10783731; doi:10.1371/journal.pone.0289572)
Supplement: S2 Table — (DOC) [file pone.0289572.s011.doc]

**Table 2. Demographic and social characteristics of the included studies**

| Study | Country | limb | Number of participants | | Mean Age (years, SD or range) | | Sex (female:male) | | Stroke side( right:left) | |
| --- | --- | --- | --- | --- | --- | --- | --- | --- | --- | --- |
|  |  |  | Exp | Ctr | Exp | Ctr | Exp | Ctr | Exp | Ctr |
| Binder et,al, 1981 | US | lower limb | 5 | 5 | No reports | No reports | No reports | No reports | No reports | No reports |
| Cristina et,al, 2021 | Spain | upper limb | 19 | 19 | 45(35-48) | 45(36-50) | 7(37%)/12(63%) | 7(37%)/12(63%) | 11 (55%)/8 (45%) | 5(62.5%)/3(37.5%) |
| Domenico et,al, 1994 | Italy | lower limb | 8 | 8 | 61.3 (12.3) | 53.5 (18.5) | 4(50%)/4(50%) | 5(62.5%)/3(37.5%) | 4(50%)/4(50%) | 5(62.5%)/3(37.5%) |
| Gülseren et,al, 2021 | Turkey | lower limb | 20 | 20 | 60.55±2.45 | 65.30±1.40 | 13(65%)/7(35%) | 12(60%)/8(40%) | 11 (55%)/9 (45%) | 12 (60%)/8(40%) |
| Lincol et,al, 1989 | UK | upper limb | 20 | 20 | 67.4±10.45 | 68.05±9.53 | 6(30%)/14(70%) | 9(45%)/11(55%) | 8(40%)/12(60%) | 6(30%)/14(70%) |
| Meryem et,al, 2012 | Turkey | upper limb | 20 | 20 | 57.90±13.32 | 60.75±12.81 | 9(45%)/11(55%) | 7(35%)/13(65%) | 10(50%)/10(50%) | 12(60%)/8(40%) |
| Paul et,al, 2013 | US | upper limb | 21 | 22 | 54 ± 12 | 57 ± 10 | 10(47.6%)/11(52.4%) | 12(54.5%)/10(45.5%) | No reports | No reports |
| Peih et,al, 2018 | Chain | lower limb | 13 | 9 | 55.5 ± 12.4 | 56.1 ± 9.0 | 9(69.2%)/4(30.8%) | 7(77.8%)/2(22.2%) | 7(50%)/7(50%) | 4(44.4%)/5(55.6%) |
| Rayegan et,al, 2014 | Iran | upper limb | 10 | 10 | 53 (9.8) | 54 (8.2) | 5(50%)/5(50%) | 6 (60%)/4(40%) | 10(100%)/0(0%) | 10(100%)/0(0%) |
| Selcan et,al, 2019 | Turkey | lower limb | 17 | 17 | 59 (18–78) | 58 (22–71) | 6 (35.3%)/11(64.7%) | 6 (35.3%)/11(64.7%) | 10 (58.8%)/7(41.2%) | 6 (35.3)/11(64.7) |

| Study | Mean stroke duration (days, SD or range) | | EMG-BFB frequence and duration | Outcome measures | Time of assessment |  |
| --- | --- | --- | --- | --- | --- | --- |
|  | Exp | Ctr |  |  |  |  |
| Binder et,al, 1981 | No reports | No reports | 3/w ,4w | Active Ankle ROM | Baseline, 4 weeks post |  |
| Cristina et,al, 2021 | 22(7) | 20(6) | 3/w,6w | Fugl-Meyer Upper Extremity,GH* Flexionº | Baseline,6weeks |  |
| Domenico et,al,1994 | 11.3 (12.6) | 8.3 (6.0) | 15 sessions | Scores of Neurological and Functional Scales、EMG-BFB Values、BI | Baseline,2months |  |
| Gülseren et,al, 2021 | 117±22.6 | 110±20.6 | 15 sessions（5/w for 3w, 20 min ) | AS,ankle dorsifexion range of motion,Brunnstrom-lower extremity values,MMAS,EMG-BF values | Baseline, 3 weeks |  |
| Lincol et,al, 1989 | 2-8w | 2-8w | 18 sessions (6 weeks) | ARA, BFM | Baseline,6、12weeks |  |
| Meryem et,al, 2012 | 199.30±222.33 | 145.40±149.97 | 5/w, 20min, 3w | UEFT、FMS、EMG-BF values、BI | Baseline,3weeks |  |
| Paul et,al, 2013 | 6.0 ± 7.4 | 12.7 ± 10.8 | 30 sessions (30 min), over 10 to 12w | UE-FMA, Stroke Impact Scale（Strength, ADL, Mobility, Hand） | Baseline,2.5-3months |  |
| Peih et,al, 2018 | 15.85 ± 11.1 | 14.8 ± 8.7 (4–28) | 18 sessions (6w, 40 minutes） | TA; NT: balance (anteroposterior sway amplitude defined by limits of stability test in dynamic posturography), walking speed, AROM；TUGT；6MWT；EPE | Baseline,1 day, 2 weeks, and 6 weeks posttraining. |  |
| Rayegan et,al, 2014 | 8.7 (10.8) | 8 (8.8) | 10 sessions, 5/w | JHFT | Baseline, 0.5months |  |
| Selcan et,al, 2019 | 95 (10–444) | 68 (10–425) | 10 sessions (5/w,2w) | Range of motion，Spasticity，Muscle strength，BI | Baseline,2 weeks,1、3months |  |

Abbreviations:JHFT = the Jebsen Hand Function Test;OT = occupational therapy; OTBF = occupational therapy and biofeedback; OTNF = occupational therapy and neurofeedback;AS = the Ashworth scale ;BS = Brunnstrom’s stage ;UEFT = the upper extremity function test ; WEAROM = active range of motion of wrist extension ;BI = the Barthel Index ; MMAS = the Modifed Motor Assessment Scale;ARA = Action Research Arm test;BFM = the Brunnstrom-Fugl Meyer test; SIS = the Stroke Impact Scale ;TA: tibialis anterior;AROM: active range of motion;
TUGT: Timed Up and Go test;6MWT: six-minute walking test;EPE: endpoint excursion; UE-FMA = the Upper Extremity Fugl-Meyer Assessment ; FMS = the Fugl-Meyer Scale; BBT =the Box-and-Block Test scores.
