# Supplementary material for: Electromyographic biofeedback therapy for improving limb function after stroke: A systematic review and meta-analysis
Source: PLoS One. 2024 Jan 11;19(1):e0289572. doi: 10.1371/journal.pone.0289572 (PMC10783731; doi:10.1371/journal.pone.0289572)
Supplement: Attachment — Submitted filename: PONE-D-23-04219_Review.pdf [file pone.0289572.s012.pdf]

Review for: Electromyographic biofeedback therapy for improving limb function after stroke: A systematic review and meta-analysis

Thank you for your work on preparing this manuscript and for providing me with the chance to review it.

The current systematic review attempts to summarize the literature on the use of electromyographic biofeedback (EMG-BFB) therapy in the rehabilitation of upper and lower limb function following stroke. A total of 10 randomized controlled trials were included in the review and meta-analysis. Reported results show a significant improvement in "limb" function following the use of EMG-BFB therapy. There were no significant differences in improvements in upper or lower limb functions separately.

While the effort undertaken by the authors to conduct the literature search and provide such comprehensive qualitative and quantitative summary of evidence is very obvious, several issues limit the usability and comprehension of the results of this work as outlined below.

Major comments:

#	Comment
1	The primary result of the analysis in the current study shows a significant improvement in limb function following the use of EMG-BFB therapy (line 210 and figure 2). However, analysis of improvements in upper and lower limbs separately show non-significant findings (line 229 and figure 3).<br><br>It remains to be explained why did the authors see the need to combine the analysis for upper and lower limbs, which could have shown the significant improvement by a mere increase in sample size. In clinical practice, therapists aim to see improvements in either upper limb function or lower limb function. Combining both limbs in the analysis is not justified.
2	The authors reported that the final synthesis of the systematic review and meta-analysis included 10 studies, the details of which are provided in supplementary table 2. However, several statements throughout the Results and Discussion sections seem to refer to studies not listed among those 10, for example, the majority of studies mentioned in the paragraph starting at line 175 (e.g., Crow et al, Cordo et al, Tsaih et al…), also, Lirio-Romero et al (line 214), Dogan-Aslan et al (line 237), Arpa and Ozcakir (line 237), just to name few. This leaves the reader wondering as to the actual number of studies included in the review and which ones they are.
3	The search strategy included reference lists of all relevant studies in previous reviews (line 112). In the Introduction, two similar meta-analyses of randomized controlled trials on EMG-BFB therapy in patients with stroke are cited; one by Moreland et al, and the other by Woodford et al (line 89). It appears that not all studies included in those two meta-analyses are included in the current manuscript.

	For example, the paper by Moreland included 8 randomized controlled trials on EMG-BFB therapy and lower limb function.<br>Also, the paper by Woodford included studies on range of motion in the ankle, knee, shoulder, and wrist joints.
4	This systematic review was limited to randomized controlled trials of EMG-BFB therapy reporting changes in upper and lower limb function in post-stroke patients. While randomized trials are known to be the gold-standard for establishing cause-and-effect relationships between interventions and outcomes, the authors repeatedly stated that the objective and findings of this study showed the association between EMG-BFB therapy and upper and lower limb dysfunction (example, lines 29, 39, 274…).<br>The wording should be changed throughout the manuscript to reflect cause-and-effect not association.
5	Among the results highlighted in the abstract (lines 44 – 46), the authors mention that EMG-BFB therapy caused significant improvement in the range of motion of glenohumeral and wrist joints. Further reading in the Results section (line 245) reveals that this statement is actually based on one study, meaning that, in accurate terms, this is not based on a meta-analysis.<br>The authors should either clarify this in the abstract or remove it from the abstract and keep it in the Results and Discussion sections where the reader can have a clear and accurate understanding of how this finding was reached.<br>Actually, the meta-analysis by Woodford et al (see comment #3 above) included randomized controlled trials that examined the range of motion at the shoulder and wrist joints which are not included in the current analysis. Including these studies, if applicable, will allow running a meta-analysis on those results.<br><br>In addition, this discussion of improvement in the range of motion does not mention whether it is active or passive range that showed the improvement.

Minor comments:

Line	Comment
Abstract (line 42) and elsewhere (e.g., line 232)	While the results reported show a significant effect size for short-term effects but not long-term ones, it is not mentioned anywhere in the text what is the timeframe meant by short- or long-term. Only in figure 4 it is mentioned that the demarcation is made by the 1-month mark. This should be clearly stated in the manuscript.
Introduction and Methods	The Introduction can be further enriched with more information about the current knowledge on the effectiveness of EMG-BFB.<br>Also, the Methods section (e.g., line 119) should include more details about the methodology of EMG-BFB. Alternatively, such details can be added about the methodology of EMG-BFB used in the included studies in Supplementary Table 2.

69 – 72	While this paragraph lists current interventions that are used and highly investigated to improve upper and lower limb function following stroke, all references cited are old.
73	Please provide a reference for this statement on interventions shortcomings.
94	The reference by the Royal College of Physicians (2004) is not listed in the References. Also, newer guidelines (2016) are available.
163	Why “more than 10 studies” when the current study included only 10?
176	The statement says “two trials took place in the US,” however references listed are three.
184	The mean age of participants in each group ranged between 45 and 67.4 years. This age represents relatively young patients in the included studies, a point which should be addressed in the Discussion section.
188	Please double check the following sentence: “Regarding the etiology of dementia, five studies included patients with lower limbs, and the other five studies included patients with upper limbs.”
191	What is the unit for the doses of interventions? Re-write the sentence.
206	What about the risk of bias in deviations from intended interventions?
230	In the sentence “results are not clinically significant,” this should be changed to statistically significant.
270	Please double check the wording in the sentence: “the probability that the effect size of phototherapy interventions was…”
281	Please double check that five studies showed a statistically significant beneficial effect on wrist and shoulder range of motion.
291	Please cite the sentence: “a previous meta-analysis evaluating EMG-BFB…”
318	Please provide a reference for this statement on impaired sensation early post stroke.
527 and 523	Legends for figures 2 and 3 are switched.
Figure 1	Last box should say “studies included in quantitative synthesis (meta-analysis)” not qualitative
Supplementary material – table 2	Please double check the numbers under stroke side in the control group in the study by Cristina et. al. The control group has 19 patients but numbers for stroke side are mentioned for 8 patients only.

Language comments:

While the manuscript has been reviewed for language, few sentences need revisiting.

Line	Sentence
60 – 61	Stroke is the leading cause of long-term disability in stroke patients…
85	EMG-BFB therapy now widely used in clinical practice…
91	In contrast, showed significant associations with improved…
188	Regarding the etiology of dementia, five studies included patients with lower limbs, and the other five studies included patients with upper limbs.

191	The mean EMG-BFB frequencies and duration of the participants ranged from…
214	We found by excluding studies (Lirio-Romero et al, 2021) significantly reduced…
320	This is a very long sentence. Please re-write.
336	...with most patients between 5 and 21 patients.
349	And due to the various scores and scales analysed were different and expressed in means, there is no method used to qualify the evidence, like GRADE.
